# Lysine Lactylation Writers: Enzymatic Versus Non-Enzymatic Mechanisms, Isomer Specificity, and Therapeutic Implications

**DOI:** 10.3390/molecules31213902

**Published:** 2026-10-29

**Authors:** Dingqin Li, Shujun Zhang

**Affiliations:** Department of Biochemistry and Molecular Biology, School of Basic Medical Sciences, Southwest Medical University, Luzhou 646000, China; lidingqin37@aliyun.com

**Keywords:** lysine lactylation, lactylation writer, L-lactylation, D-lactylation, non-enzymatic lactylation, lactyl-CoA, therapeutic targeting

## Abstract

Lysine lactylation (Kla) is a key modification linking cellular metabolism to epigenetic regulation, but its chemical nature is more complex than initially appreciated. It comprises two generation pathways differing in chirality and catalytic logic: enzymatic L-lactylation (KL-la) and non-enzymatic D-lactylation (KD-la). In 2024, breakthroughs in enantiomer-resolved techniques clarified their regulatory origins: KL-la is mainly driven by glycolytic flux, whereas KD-la depends on loss of function of the glyoxalase system. This chemical duality means that “who writes lactylation” is no longer a simple question of enzyme assignment but a highly context-dependent judgment. This review critically assesses functional evidence for the lactyl-CoA-dependent KAT family, the lactyl-AMP-dependent AARS family, and the non-canonical catalytic activity of HDACs. It analyzes in particular the substrate concentration paradox, the enantiomeric ambiguity of antibodies marketed as pan-Kla, and the causal confounding arising from shared enzymatic machinery between Kla and acetylation. On this basis, it constructs an integrated regulatory model centered on site-level net flux. Therapeutically, strategies targeting lactylation pathways show preliminary translational promise, but most interventions still perturb acetylation programs simultaneously. Future breakthroughs may depend on developing flux measurement tools, standardizing enantiomer-resolved detection, and shifting from single-target inhibition to strategies based on modification-profile stratification.

## 1. Introduction

The role of L-lactate in biology has undergone a paradigm shift from a terminal product of glycolysis to an energy substrate, and then to an epigenetic donor [1,2,3,4,5]. In 2019, Zhang et al. demonstrated by mass spectrometry and isotope tracing that L-lactate can be covalently attached to the ε-amino group of histone lysine via lactyl-CoA under p300 catalysis, forming Kla and directly regulating transcription [5,6,7]. This finding elevated L-lactate from a metabolic end product to a direct chemical donor for chromatin states, providing for the first time a L-lactate-centered molecular pathway for metabolism–epigenetics coupling [6,7,8]. However, the chemical space of Kla has been more complex than that of acetylation from the outset: L-lactate is chiral, and L-lactylation (KL-la), D-lactylation (KD-la) derived from the glycolytic byproduct methylglyoxal (MGO), and N-ε-carboxyethyllysine (Kce) share the same nominal mass shift (+72.021 Da) and are indistinguishable by routine detection [7,9,10]. In 2024, the teams of Di Zhang and Yingming Zhao used chiral derivatization HPLC combined with isomer-specific antibodies to systematically distinguish KL-la, KD-la, and Kce on histones for the first time, demonstrating that KL-la is the dominant isomer under physiological conditions and is selectively driven by glycolysis; KD-la and Kce increase significantly only upon GLO2 loss or MGO accumulation [7,9]. This work not only provided a chemical basis for isomer resolution in lactylation research but also directly undermined the reliability of previous conclusions that used these reagents as if they were pan-Kla antibodies [7,9,11].

**Figure 1:**
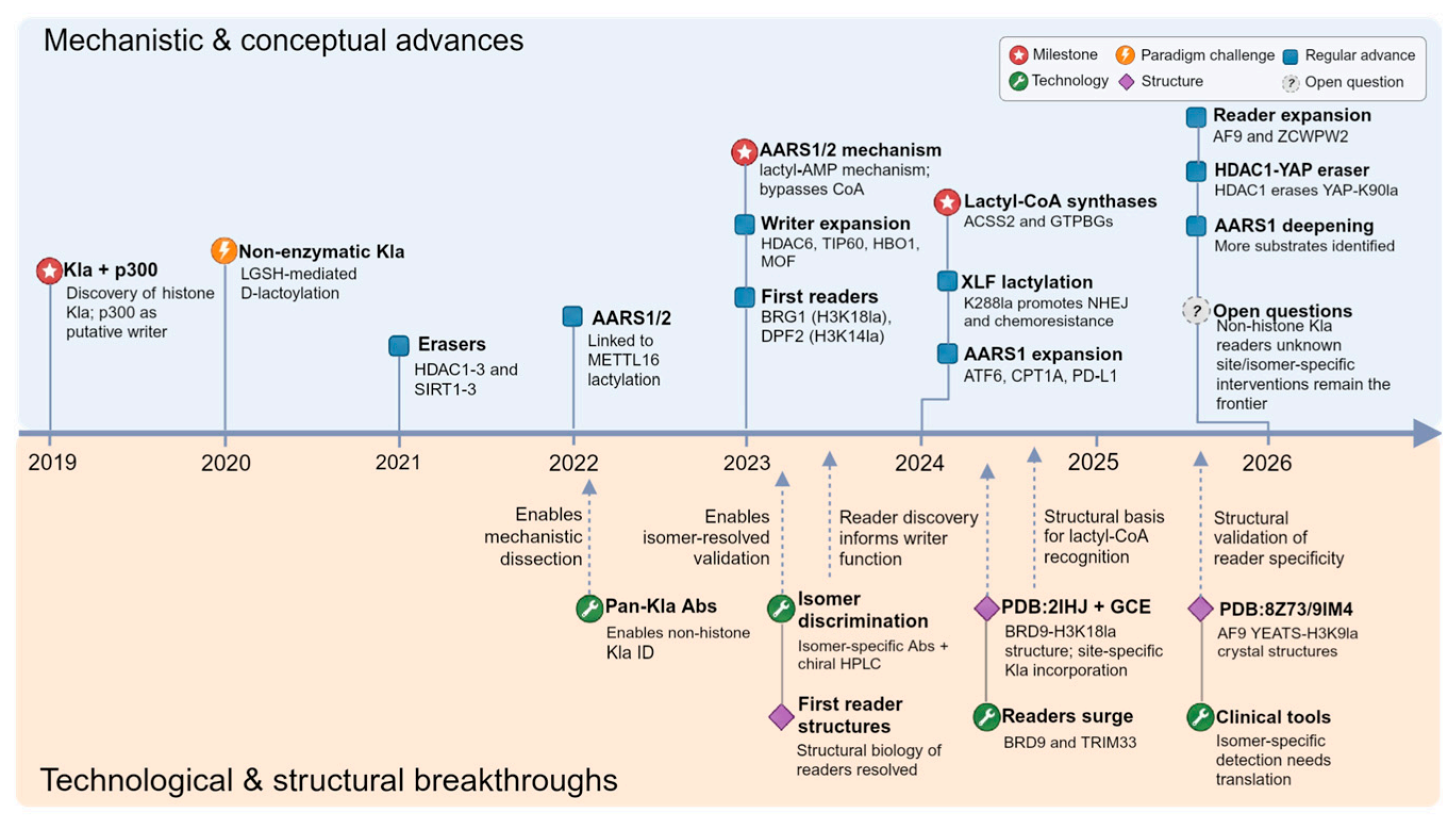
Timeline of mechanistic, conceptual, and technological breakthroughs in lactylation research (2019–2026). Created in BioRender. Zhang, S. (2026) https://BioRender.com/5ep7qt0 accessed on 20 October 2026.

With the widespread use of antibodies marketed as pan-Kla (in practice, anti-L-lactylation antibodies) and quantitative mass spectrometry, the lactylation modification landscape rapidly expanded from histones to non-histones [10,12]. Functional Kla sites have now been identified in metabolic enzymes (e.g., PDHA1, CPT2), DNA damage repair proteins (MRE11, NBS1), RNA-modifying enzymes, and transcriptional regulators [13,14]. This expansion has brought a core question to the forefront: who is “writing” lactylation? p300/CBP was first reported to catalyze histone lactylation using lactyl-CoA, and subsequently acetyltransferases such as GCN5, HBO1, TIP60, and MOF were also found to possess lactylation activity in vitro or in cells (Figure 1) [6,15,16,17,18,19,20]. More complexly, in 2024 AARS1/2 were identified as lactyl-CoA-independent lactyltransferases that directly attach L-lactate to lysine residues via a lactyl-AMP intermediate [21]. Subsequently, ACSS2 and GTPSCS were shown to act as bona fide lactyl-CoA synthetases, providing upstream substrates for enzymatic L-lactylation [15,22]. Moreover, Class I HDACs (HDAC1–3) were found to reverse their canonical deacetylation direction and catalyze lysine lactylation under specific metabolic conditions [23]. This finding further blurred the traditional functional boundary between writers and erasers.

However, the rapid expansion of candidate writers has not brought consensus; instead, it has exposed a series of evidence-level problems [6,24]. The conclusions of most studies rely heavily on in vitro experiments with recombinant proteins or on enrichment signals from anti-L-lactylation antibodies; apart from a few methodological benchmark studies, site-resolved stoichiometric data are lacking [6,9]. Some intracellular evidence has not distinguished L- from D-lactylation, or even excluded MGO-derived non-enzymatic modifications [9]. Different laboratories have drawn inconsistent conclusions regarding whether the same protein, such as p300 or AARS1, possesses physiological lactyltransferase activity [6,25,26]. Given that these discrepancies cannot be resolved by a single experimental paradigm, recent reviews advocate a framework of tiered evidence assessment, advancing lactylation research from “detection equals establishment” to the standard of “site-resolved, isomer-defined, and causally testable” [27]. Therefore, it is necessary to re-examine the current state of lactylation writer research on the basis of critical evidence assessment [27]. Under the chemical premise of isomer resolution, this review grades the evidence for candidate writers, dissects the methodological roots of controversies, and builds a testable regulatory model and therapeutic translation framework on this basis. A comprehensive list of abbreviations used throughout this review is provided in Table S1.

## 2. The Duality of Lactylation Generation Mechanisms

### 2.1. Enzymatic L-Lactylation: Two Parallel Activation Strategies

**Figure 2:**
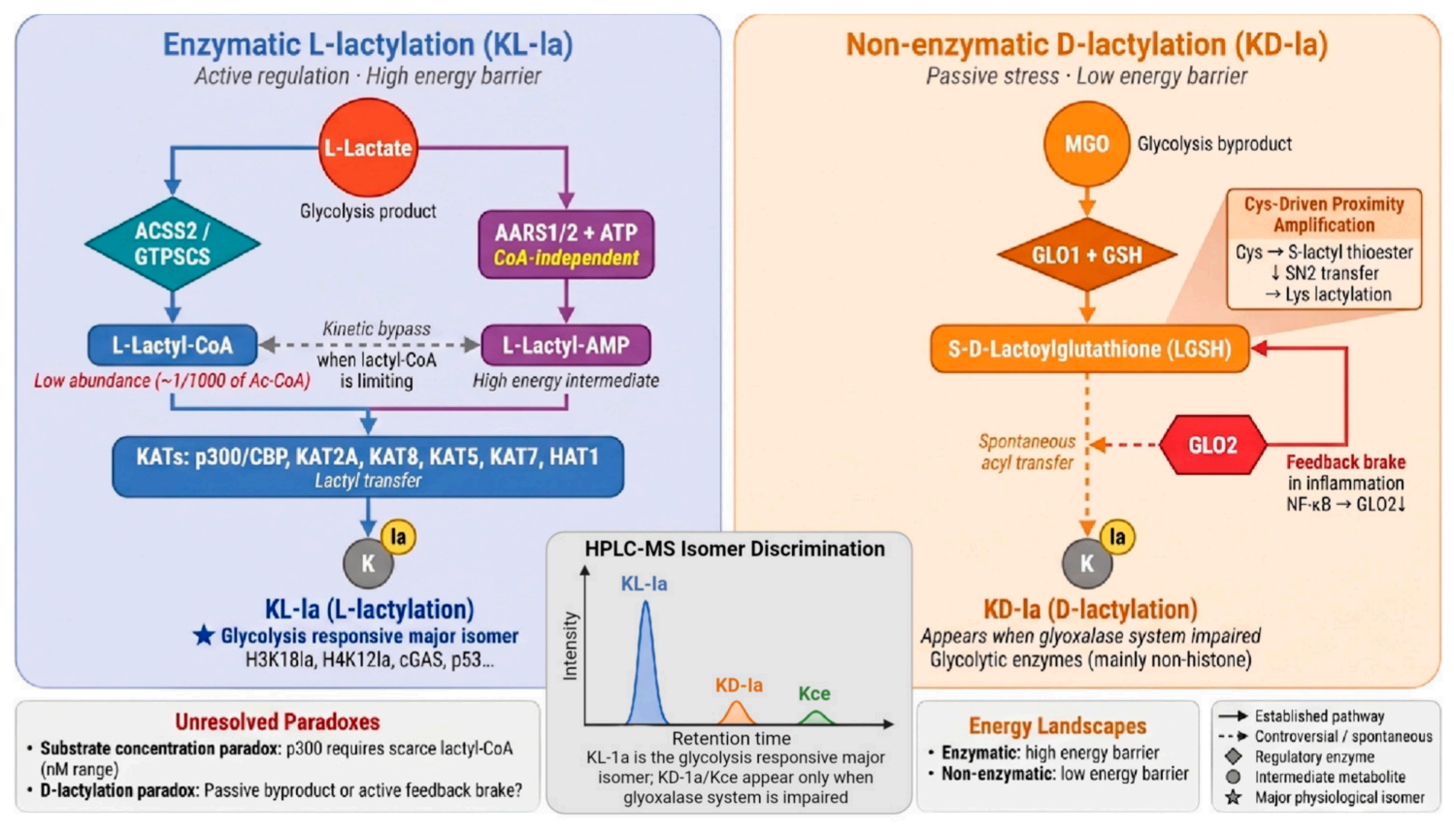
Mechanistic dichotomy of enzymatic L-lactylation and non-enzymatic D-lactylation: energy landscapes, isomer discrimination, and unresolved paradoxes. The red downward arrow (T-bar) indicates the downregulation or inhibition of GLO2 under specific conditions. Non-histone substrates such as p53 are also representative targets of KL-la, as indicated at the bottom left. The HPLC-MS inset illustrates the retention time (horizontal axis) and intensity (vertical axis) for isomer discrimination. Created in BioRender. Zhang, S. (2026) https://BioRender.com/p9goq7u accessed on 20 October 2026.

L-lactate lacks the electrophilicity required for direct lysine acylation and must first be activated into a high-energy intermediate [6]. Cells have evolved two parallel strategies: the thioester pathway (lactyl-CoA) and the phosphoanhydride pathway (lactyl-AMP) (Figure 2) [27,28].

The lactyl-CoA pathway is chemically analogous to acetylation: after CoA thioesterification of L-lactate, acyl transfer is completed by proteins with lysine acyltransferase activity [15,29]. Compared with acetyl-CoA, however, lactyl-CoA faces a fundamental physicochemical constraint: its steady-state intracellular concentration is extremely low. Quantitative analyses show that lactyl-CoA abundance in mammalian cells is only about 1/1000 that of acetyl-CoA [26]. The Km of p300 for lactyl-CoA is in the micromolar range, which could in theory support catalysis under locally enriched conditions, but the in situ concentration of nuclear lactyl-CoA has never been directly measured [30]. This paradox must be evaluated in terms of the local rather than the bulk lactyl-CoA pool. Lactyl-CoA metabolism is subcellularly compartmentalized: nuclear GTPSCS and ACSS2 function as dedicated lactyl-CoA synthetases and generate a nuclear lactyl-CoA pool that is physically coupled to histone lactyltransferase complexes such as p300/CBP and KAT2A [15,22,31,32]. Consequently, whole-cell lactyl-CoA abundance—typically reported as orders of magnitude lower than acetyl-CoA—should not be used as a standalone argument against p300/CBP-mediated lactylation. The functionally relevant parameter is the local lactyl-CoA concentration delivered to chromatin-associated acceptor lysines, which may be substantially higher than bulk measurements imply. This local-pool model provides a plausible resolution to the substrate concentration paradox; however, quantitative flux partitioning data are still lacking [27,28].

Zhu et al. (2025) showed that ACSS2 can generate lactyl-CoA at L-lactate concentrations of 5–20 mM and forms a physical complex with KAT2A to catalyze H3K14la and H3K18la [15,32]. However, the apparent selectivity ratio of ACSS2 for acetate versus L-lactate has not been systematically determined, and its nuclear translocation depends on S659 phosphorylation (regulated by AMPK/AKT), suggesting that this lactylation function is a conditional event rather than constitutive activity [33]. GTPSCS (succinyl-CoA synthetase α subunit, SUCLG2) is a more unexpected candidate lactyl-CoA synthetase [22]. This TCA cycle enzyme translocates to the nucleus in glioma cells and complexes with p300, catalyzing lactyl-CoA generation from L-lactate with a Km of approximately 15.3 mM [22,34]. This Km value matches L-lactate concentrations in the solid tumor microenvironment, but how it is structurally compatible with canonical succinyl-CoA synthetase activity—whether through substrate promiscuity or a conformational switch—remains unclear [4]. Importantly, the original study published in Cell Metabolism provided a key rationale and experimental evidence for GTPSCS-mediated lactyl-CoA synthesis rather than succinyl-CoA synthesis [22]. Specifically, competition experiments using physiological concentrations of succinate and L-lactate showed that L-lactate, rather than succinate, serves as the substrate for GTPSCS under the relevant conditions [22]. In the TCA cycle, ATP-specific succinyl-CoA synthetase (ATPSCS) is the preferred enzyme, whereas GTPSCS is not [22,28]. In addition, GTP and ATP concentrations critically influence whether GTPSCS or ATPSCS carries out the relevant reaction [22]. Together, these findings provide a rationale for context-dependent lactyl-CoA synthesis by GTPSCS and help explain its nuclear function [4,22].

The lactyl-AMP mechanism of AARS1/2 bypasses the lactyl-CoA bottleneck [35]. Li et al. (2024) demonstrated that AARS1/2 bind L-lactate with micromolar affinity (AARS1 Kd = 16.7 μM, AARS2 Kd = 7.5 μM) and, after ATP-dependent generation of lactyl-AMP, transfer it to lysine [21]. AARS1 is cytoplasmic and lactylates non-histone targets such as YAP, TEAD1, and p53 (K120/K139, inhibiting DNA binding) (Figure 2) [21,25,36]; AARS2 is mitochondrial and lactylates PDHA1 (K336) and CPT2 (K457/K458), and its gain-of-function mutations promote premature ovarian failure through hyperlactylation [14,37]. However, recent genetic and biochemical studies show that AARS1/2 loss does not reduce global Kla, and that lactylation and acetylation are mediated by different enzymes [23,38,39]. A more accurate characterization may be that AARS1/2 are highly selective L-lactate sensors and site-specific lactyltransferases rather than pan-Kla writers—their L-lactate-binding pocket mediates specific recognition, L-lactate binding triggers conformational activation, and their lactyltransferase activity is independent of canonical editing functions [40].

### 2.2. Non-Enzymatic D-Lactylation

D-Lactylation begins with MGO, an unavoidable byproduct of non-enzymatic dephosphorylation of triose phosphates during glycolysis [41]. Adjacent cysteine residues can promote Nε-carboxyalkylation of lysine by MGO via a hemithioacetal intermediate (Figure 2) [42]. GLO1 condenses MGO with GSH to form LGSH, and GLO2 hydrolyzes LGSH to D-lactate [10,43]. The thioester bond of LGSH has intrinsic acyl transfer potential and can modify adjacent lysines through intramolecular S→N transfer to form KD-la [10,44]. GLO2 is the rate-limiting enzyme: when its activity is normal, the half-life of LGSH is extremely short and the KD-la generation rate is low [10,43]; when GLO2 is downregulated, LGSH accumulates and provides a time window for spontaneous acyl transfer [44]—NF-κB signaling specifically downregulates GLO2 through TTP-mediated mRNA decay, leading to cytosolic accumulation of SLG, and D-lactylation of RelA K310 attenuates NF-κB transcriptional activity to restore immune homeostasis [44]. D-Lactylation exists as a metabolic regulatory mechanism in both eukaryotes and prokaryotes, and mouse testicular histones carry both L- and D-lactylation modifications [45,46]. Host-derived D-lactate is generated through the glyoxalase pathway, participates in immune response regulation in macrophages and brain tissue, and is associated with inflammation resolution and neuroimmune communication [47,48].

The team of Xuetao Cao (2025) revealed an efficiency-amplifying mechanism of this pathway: adjacent cysteine first forms an *S*-D-lactylated thiol intermediate with LGSH, which then modifies proximal lysine via S→N transfer, substantially broadening the substrate space [44]. In innate immune activation, NF-κB downregulates GLO2 through TTP-mediated mRNA decay, establishing a positive feedback loop wherein immune activation downregulates GLO2, leading to LGSH accumulation and consequently elevated D-lactylation [44]. Therapeutically, the GLO2 inhibitor DiFMOC-G shows efficacy in acute inflammation and IBD models, with its mechanism interpreted as deliberately elevating D-lactylation to trigger immunomodulatory output [44]. However, the substrate spectrum, reversibility, and functional mechanisms of D-lactylation are almost entirely unknown. Given its extremely weak reversibility, the cumulative effects and safety of long-term pharmacological elevation remain to be evaluated [44,49].

### 2.3. Methodological Turning Point in Isomer Resolution

The similarity in chemical outcome between enzymatic KL-la and non-enzymatic KD-la (both add a lactyl group to lysine, with a mass shift of 72.021 Da) has long made them indistinguishable by routine mass spectrometry and antibody detection [6,10,50]. Commercial anti-Kla antibodies available before 2024, including the first PTM Bio reagents, were raised against L-lactyl peptides and are therefore best described as anti-L-lactylation antibodies, although they were not specifically labelled as such. Their reactivity toward D-lactylation and Kce was rarely validated by chiral methods, and they should not be treated as validated enantiomer-agnostic pan-Kla antibodies. Therefore, the vast majority of lactylation studies published between 2019 and 2023, based on antibodies marketed as pan-Kla or on mass spectrometry data without chiral resolution, may have reported a weighted mixed signal of KL-la, KD-la, and Kce [6,10,51,52]. In 2024, the orthogonal resolution strategy of the teams of Di Zhang and Yingming Zhao resolved this methodological bottleneck, achieving enantiomer-resolved quantification at the histone site level for the first time [9,53]. This established an independent regulatory model in which “KL-la is driven by glycolytic flux, while KD-la is driven by loss of glyoxalase function” [9,10,52]. In 2025, mouse testicular data further revealed site-level chiral preference [46,54], but whether this reflects abnormal local MGO concentrations, differences in GLO2 expression, or selective de-modification mechanisms for D-lactylation still requires further investigation [9,10,44,55].

## 3. Candidate Lactylation Writers and Their Classification

All enzymes currently reported to possess lactylation catalytic activity are “moonlighting” participants in other acylation modifications or metabolic pathways [56]. According to catalytic mechanism, they can be classified into lactyl-CoA-dependent (KAT family) [6], lactyl-AMP-dependent (AARS family) [21], reversible catalytic (HDAC family) [23], and prokaryotic writers (YiaC) [24]. Table 1 summarizes the evidence profiles for each candidate enzyme.

### 3.1. Lactyl-CoA-Dependent KAT Family

Among lactyl-CoA-dependent writers, p300/CBP was the first enzyme demonstrated to catalyze histone lactylation using lactyl-CoA [6,22,57]. The lactyltransferase activity of p300/CBP is supported by multiple independent experimental approaches, not solely by in vitro enzymology [6,22,57]. In 2019, Zhao and colleagues showed that p300 overexpression or knockdown in HEK293T cells altered global histone lactylation, and cell-free recombinant chromatin assays confirmed that p300 directly deposits lactyl groups on histones [6]. Subsequent studies extended these findings using p300 knockdown in HCT116 and HEK293T cells, p300 interference in macrophages, and overexpression of p300/CBP alongside other acyltransferases [6,57]. Functionally, p300 knockdown reduces hnRNPA1 lactylation, whereas knockdown of GCN5, PCAF, or AARS1 does not produce the same effect, supporting a degree of substrate selectivity [58]. Beyond histones, CBP/p300 has been reported to lactylate non-histone proteins including the DNA-repair factor MRE11 at K673 in response to DNA damage, CRYAB at K92, KU70 at K317, and HMGB1 [59,60,61,62]. Together, these genetic, overexpression, knockdown, and biochemical data establish p300/CBP as a bona fide lactyltransferase acting on a range of histone and non-histone substrates. The unresolved question is therefore not whether p300/CBP can write Kla, but how its site-specific and context-dependent contribution is quantitatively partitioned relative to parallel pathways. The Km of p300 for L-lactyl-CoA is in the micromolar range, indicating that it possesses the kinetic basis to catalyze lactylation when L-lactate concentrations rise [6]. Cu^2+^ can selectively inhibit lactylation by electrostatically binding histone Glu95 [63]. However, a 2025 study found that Class I HDACs (1/2/3) can directly catalyze lactylation and are the major drivers maintaining global basal Kla levels in cells. This finding suggests that p300/CBP-dependent lactyl-CoA transfer may not be the dominant pathway for global Kla formation, although its role under specific substrates or biological contexts still needs further definition [23]. Subsequently, Liu et al. (2025) found that GTPSCS can translocate to the nucleus and form a complex with p300, generating lactyl-CoA in situ near chromatin and thereby driving H3K18la; this “local substrate supply” model provides a spatial mechanistic explanation for p300/CBP-mediated lactylation writing in the nucleus [22,64]. KAT2A/GCN5 participates in lactylation by forming a physical complex with ACSS2: Zhu et al. (2025) used co-immunoprecipitation and proximity ligation assays to confirm that ACSS2 directly binds KAT2A, with ACSS2 converting L-lactate to lactyl-CoA and KAT2A utilizing this substrate to catalyze H3K14la and H3K18la, together constituting an efficient local catalytic unit [15,65]. KAT8/MOF has been reported to exhibit a preference for lactylation, and lactylation of its representative substrate eEF1A2 at K408 promotes translational elongation and proliferation in colorectal cancer cells [18,66]. In addition, KAT2B/PCAF was identified as the lactyltransferase for PGK1 K353, participating in liver fibrosis progression [67]; KAT5/TIP60 lactylates NBS1 and participates in DNA damage response and chemoresistance [17]; KAT7/HBO1 possesses lactylation catalytic activity [16]; and HAT1 substrates include RPA1 K88la [68] and PD-L1 K75/K178la, the latter enhancing PD-L1 stability through a glycosylation-dependent mechanism and promoting immune evasion [68,69]. Notably, a non-enzymatic D-lactylation mechanism has also been revealed: the GLO2 substrate SLG accumulates under inflammatory conditions and non-enzymatically modifies adjacent lysine through cysteine-mediated S→N transfer, and RelA K310la can feedback-inhibit NF-κB activity [44,70]. These KAT family members and non-enzymatic pathways exhibit lactylation writing capacity under specific metabolic conditions and substrate contexts, but their activity strength and physiological contribution need to be evaluated in the context of specific cellular environments.

### 3.2. Lactyl-AMP-Dependent AARS Family

AARS1/2 do not depend on lactyl-CoA; instead, they directly utilize ATP to activate L-lactate to lactyl-AMP, which then completes lysine acyl transfer [21,26,71]. The core findings for AARS1 and AARS2 each came from 2024 studies, which demonstrated that AARS1 can lactylate multiple lysine sites on YAP, enhancing YAP–TEAD interaction and downstream transcription [26]; lactylation of the p53 DNA-binding domain can weaken its tumor-suppressive activity [25,72]. AARS2, in contrast, lactylates PDHA1 and CPT2 in mitochondria, affecting pyruvate oxidation and fatty acid oxidation [14,37]. In addition, AARS2-mediated H3K18la has been reported to participate in ferroptosis regulation in intestinal ischemia–reperfusion injury [73].

### 3.3. Non-Canonical Catalytic Functions of the HDAC Family

HDAC family members are traditionally regarded as deacetylases, but recent studies have found that some members can catalyze lactylation under specific conditions, exhibiting non-canonical writing activity [23]. In 2025, Gonzatti et al. reported that Class I HDACs (HDAC1–3) can catalyze lactylation under high-L-lactate conditions, and their Zn^2+^-water/acid catalytic system has no intrinsic restriction on the direction of acyl donor transfer; the net reaction direction is determined by the mass action law of substrates and products [23]. HDAC6 provides another striking example: its canonical substrate α-tubulin K40 acetylation site can also be lactylated, suggesting a “substrate anchoring–acyl promiscuity” model, in which the enzyme first anchors through non-catalytic interactions with the substrate protein and then decides whether to transfer an acetyl or lactyl group based on the local acyl donor concentration [74]. Experimental evidence shows that HDAC6 can catalyze α-tubulin K40 lactylation in vitro, and cellular studies support its function as a lactyltransferase on microtubules [74]. These findings expand the functional repertoire of the HDAC family, transforming it from a purely “eraser” role into a bifunctional enzyme that may bidirectionally regulate acylation modifications according to the metabolic environment, but the directionality and physiological relevance of its writing activity still require further validation in more cellular contexts [65,75].

### 3.4. Prokaryotic Writers

In *E. coli*, YiaC was identified as a lysine lactylase, while CobB (a Sir2 homolog) functions as a delactylase; together they regulate multiple endogenous Kla sites [24]. The prokaryotic system provides a simpler evolutionary model: YiaC has low sequence homology with mammalian KAT families, and the existence of its lactylation activity suggests that the enzymatic capacity for lactylation may have emerged before the expansion of the KAT family [76].

**Table 1:** Lactylation writers and their evidence profiles.

Writer	Catalytic Mechanism	Representative Substrate/Site	Evidence Level	References
p300/CBP	Lactyl-CoA-dependent	H3K18la, H3K9la, H4K12la; MRE11 K673la, CRYAB K92la, KU70 K317la, hnRNPA1	High	[6,22,57,58,59,60,61,62]
KAT2A/GCN5	Lactyl-CoA-dependent	H3K14la, H3K18la	High	[15]
KAT2B/PCAF	Lactyl-CoA-dependent	PGK1 K353,TBX5	Low	[72,77]
KAT5/TIP60	Lactyl-CoA-dependent	NBS1, H4K12la (synergistic with KAT8)	Medium	[17,78]
KAT7/HBO1	Lactyl-CoA-dependent	H3K9la	Medium	[16]
KAT8/MOF	Lactyl-CoA-dependent	eEF1A2 K408la	Medium	[18]
HAT1	Lactyl-CoA-dependent	RPA1 K88la; PD-L1	Low	[68,69]
AARS1	Lactyl-AMP-dependent	YAP, p53, TEAD1	High	[25,26]
AARS2	Lactyl-AMP-dependent	PDHA1, CPT2, cGAS	High	[14,37]
HDAC1–3	Reversible reaction (eraser and writer)	Histones and non-histone proteins	Medium	[23,51]
HDAC6	Substrate anchoring	α-tubulin K40	Low	[74]
YiaC	Prokaryotic	*E. coli* Kla sites	Medium	[24]
NAA10	Lactyl-CoA-dependent	NSUN2 K508	Preliminary	[79]
KAT6B	Lactyl-CoA-dependent	TNFRSF25 K396	Preliminary	[80]
ACAT1	Lactyl-CoA-dependent	Lactate metabolic regulator	Candidate	[81]
GNAT13 (S. mutans)	Prokaryotic GNAT superfamily	RpoA K173	Preliminary	[82]

Note: Evidence levels are defined as follows: High, consistent in vitro kinetics, intracellular site validation, and functional data; Medium, partial fulfillment of the above criteria; Low, single report or indirect evidence. Evidence for p300/CBP includes in vitro enzymology, overexpression, knockdown, and genetic perturbation across multiple cell types. For HDAC1–3, ref. [23] supports lactylation writing activity, and ref. [51] supports de-lactylation/eraser activity.

## 4. Critical Assessment of Writer Controversies and Methodological Limitations

### 4.1. p300/CBP

As the first identified histone lactylation writer, p300/CBP has been extensively validated by in vitro enzymology, cellular knockdown/overexpression, and genetic perturbation experiments [6]. These studies provide strong evidence that p300/CBP can use lactyl-CoA to catalyze histone lactylation. However, important quantitative questions remain. The catalytic efficiency of p300/CBP for lactyl-CoA is lower than that for acetyl-CoA, and the intracellular concentration of lactyl-CoA is approximately 1000-fold lower than that of acetyl-CoA [13,29,83]. This substrate availability gap does not negate p300/CBP function, but it raises the unresolved question of how p300/CBP contributes to site-specific Kla under physiological conditions. A further complication comes from pharmacological inhibition: in LDHA/B double-knockout cells, exogenous L-lactate restores Kla levels, whereas the p300/CBP inhibitor A485 does not fully block this restoration [23]. This result suggests that p300/CBP-independent lactylation pathways may exist; however, it should not be interpreted as evidence that p300/CBP is not a major lactyltransferase, because A485 may have incomplete target engagement, and compensatory mechanisms or additional writers may operate. It is also important to note that the evidence for p300/CBP as a lactyltransferase rests on genetic and biochemical experiments—overexpression, knockdown, and in vitro reconstitution—not on A485 sensitivity alone [6,22,23,57]. The local substrate channeling model further reconciles the apparent paradox: nuclear GTPSCS and ACSS2 generate lactyl-CoA in situ, and this locally produced pool can be directly used by chromatin-associated p300/CBP before dilution into the bulk acyl-CoA pool [15,22]. Low whole-cell lactyl-CoA abundance therefore does not exclude a quantitatively significant contribution of p300/CBP at specific chromatin loci. The discovery of AARS1/2 provided a lactyl-CoA-independent mechanism that can act in parallel with p300/CBP, rather than replacing it [21]. Thus, p300/CBP is well established as a lactyltransferase, but its quantitative, site-specific, and context-dependent contribution to global Kla remains to be determined.

### 4.2. AARS1/2

The identification of AARS1/2 in 2024 added a lactyl-CoA-independent route to lactylation: AARS1/2 directly activate L-lactate to lactyl-AMP and then transfer the lactyl group to lysine [21]. This mechanism can operate in parallel with, rather than replace, lactyl-CoA-dependent lactylation. Multiple genetic knockout experiments support a critical role for GTPSCS- or ACSS2-mediated lactyl-CoA synthesis in lactylation [15,22]. In contrast, genetic loss-of-function evidence for AARS1/2 in vivo remains limited, and AARS1/2 knockdown experiments require careful controls because these enzymes are essential aminoacyl-tRNA synthetases; reduced AARS1/2 protein abundance may secondarily affect global translation and cell fitness. Therefore, quantification of AARS1/2 protein levels, rescue experiments, and controls for global translation are essential, and conclusions based solely on knockdown without such controls may be misleading.

In vitro biochemical analyses showed that the dissociation constants (Kd) of AARS1 and AARS2 for L-lactate are 16.7 μmol/L and 7.5 μmol/L, respectively, suggesting that AARS2 has a higher affinity for L-lactate [21]. Molecular docking experiments identified the binding sites of L-lactate within the catalytic domain of AARS1 (R77, M100, W176, V218, D239); mutation of all these sites completely abolished lactyltransferase activity [84]. However, recent studies have revised the “pan-writer” model of AARS1/2: AARS1/2 are not universal Kla writers, lactylation and acetylation are mediated by different enzymes, and their substrate spectrum appears more concentrated on non-histone proteins such as YAP, p53, PDHA1, CPT2, and cGAS [48,85]. Their contribution to histone lactylation therefore requires further definition. More importantly, the functional mode of AARS1/2 is influenced by the relative abundance of L-lactate and alanine; alanine acts as a natural competitive inhibitor occupying the same binding pocket [86]. Under conditions of lactate accumulation caused by the Warburg effect, AARS1/2 may shift from their canonical aminoacyl-tRNA synthetase function toward lactyltransferase activity [40]. This substrate competition mechanism suggests that metabolite concentrations can directly determine the functional bias of the enzyme, rather than relying solely on conformational changes or post-translational modifications of the enzyme itself [86].

### 4.3. HDACs

The role of Class I HDACs (HDAC1–3) in lactylation regulation is particularly complex. On the one hand, HDAC1-3 are major de-lactylases, HDAC3 exhibits prominent efficiency in removing both L- and D-lactylated lysine in vitro [51]. On the other hand, a 2025 study found that they can reverse the reaction direction under specific conditions to catalyze Kla formation [23]. Their net functional direction may depend on the L-lactate concentration gradient: under low-L-lactate conditions, they favor de-lactylation, whereas under high-L-lactate conditions, they may shift toward the writing direction [75,87]. This condition dependence makes the “writer” identity of HDAC1-3 context-dependent and unsuitable for simple assignment to a fixed category [65]. The situation for HDAC6 differs. In vitro studies suggest that HDAC6 possesses de-lactylase activity, but cellular evidence mainly supports its function as an α-tubulin K40-specific lactyltransferase [74,88]. Classifying HDAC6 as a “bifunctional enzyme” still lacks sufficient validation of de-lactylase activity [65]. The study by Sun et al. (2024) explicitly pointed out that HDAC6 displays dual enzymatic activity in vitro but mainly functions as a lactyltransferase in cells [74]. This discrepancy highlights the decisive influence of in vitro versus in vivo conditions (such as substrate concentration, cofactor availability, and protein complex formation) on the functional direction of the enzyme [65].

### 4.4. Methodological Blind Spots and the Challenge of Isomer Discrimination

The enantiomeric ambiguity of antibodies marketed as pan-Kla is the most serious methodological problem [9,46,61,70,89]. In practice, the antibodies sold before 2024, including the original PTM Bio pan-lactylation reagents, were raised against L-lactyl peptides and should be regarded as anti-L-lactylation antibodies. Their enantiomer specificity was seldom rigorously validated by chiral resolution, and they cannot be assumed to report D-lactylation or Kce equivalently. Early “Kla” signals obtained with these reagents should therefore be interpreted primarily as L-lactylation signals and require re-evaluation with isomer-resolved methods [9,46,61,70,89]. KL-la, KD-la, and Kce have the same molecular weight and similar structures, making them difficult to distinguish by routine mass spectrometry and immunoassays [9,46]. It was not until 2024 that the teams of Di Zhang and Yingming Zhao resolved this problem through an orthogonal discrimination technology [9]. However, this technical platform has not yet been widely applied in non-histone lactylation research [84]. A large number of reported “lactylation sites” still need to be re-validated by chiral resolution methods [9,89]. Early studies mixed modification events of different chemical nature at the “total Kla” level, making data from different laboratories difficult to compare directly [9,52]. The shared enzymatic machinery between Kla and Kac is another important source of interference [23,89]. p300/CBP and HDAC1-3 simultaneously regulate acetylation and lactylation; when these enzymes are inhibited or activated, both modifications may change simultaneously, making it difficult to determine causality [23,84]. A 2026 review explicitly stated that at the same lysine site, Kla and Kac, as well as other acylation modifications, represent competing modification states rather than simple additive effects [89,90]. Therefore, an increase in H3K18la or total Kla signal should not be simply interpreted as a fixed transcriptional output; it must be comprehensively evaluated in combination with chromatin regions, neighboring modification combinations, and target gene expression profiles [89,91].

### 4.5. Lag in Reader Research

Readers are the weakest link in the lactylation regulatory network. Early on, only three Kla readers were identified: BRG1, DPF2, and TRIM33 [92,93,94]. In 2025–2026, BRD9, AF9, and ZCWPW2 were successively validated as readers, and multiple candidates have been reported (Table 2) [95,96,97]. Among them, AF9 recognizes H3K9la through its YEATS domain, and its low-affinity recognition has been interpreted as a threshold-sensing mechanism: effective recruitment occurs only when local lactylation accumulation reaches a certain threshold, possibly a “gating” design to prevent noise signals from triggering transcriptional responses [96]. ZCWPW2 can simultaneously recognize H3K18la and H3K9me3, making it the first reported lactylation–methylation dual-mark reader [97]. However, all the newly identified readers require more independent validation, and readers for non-histone Kla remain rarely reported, constraining systematic understanding of lactylation functional output [98].

### 4.6. Critical Examination of Evidence Hierarchy

**Figure 3:**
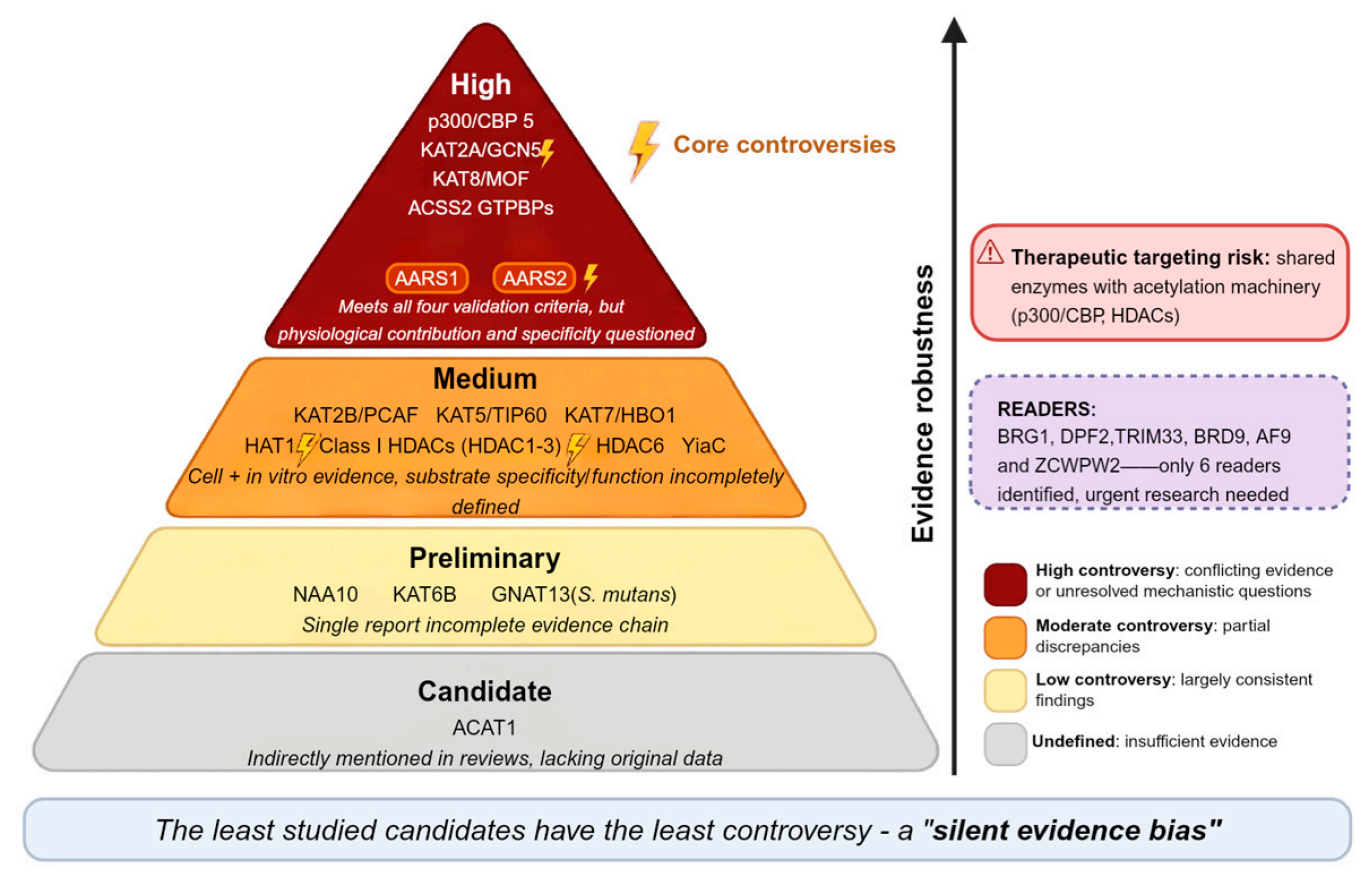
An evidence hierarchy of the lactylation machinery: core controversies, therapeutic targeting risks, and the “silent evidence bias.” The pyramid categorizes reported and candidate writers into High, Medium, Preliminary, and Candidate tiers based on experimental robustness. Note: GNAT13 indicates a prokaryotic GNAT-superfamily lactyltransferase annotated in *Streptococcus mutans*, rather than a mammalian HGNC-standard gene symbol. Created in BioRender. Zhang, S. (2026) https://BioRender.com/d01wu1m accessed on 20 October 2026.

Identifying a writer typically requires progressive evidence: cellular functional validation, in vitro enzyme activity assays, substrate site validation, and physiological function validation [4]. p300/CBP and AARS1/2 are more controversial not because the evidence is insufficient, but because they have been studied more deeply, and their limitations have been most fully exposed (Figure 3) [23,39]. In contrast, some candidate writers appear “uncontroversial” largely because they have been insufficiently studied [104]. At the same time, evidence strength and physiological contribution are not the same concept. A well-validated catalytic function does not necessarily mean that an enzyme is the major contributor to Kla under physiological conditions [4]. For p300/CBP, in vitro catalytic activity is well established, but its inefficient utilization of lactyl-CoA and the incomplete blocking effect of A485 leave its quantitative contribution unresolved [13,23]. This uncertainty should not be taken as evidence against p300/CBP function; rather, it indicates that pharmacological inhibition alone cannot define physiological flux. Similarly, AARS1/2 provide a lactyl-CoA-independent route, but their in vivo genetic evidence remains limited compared with that for lactyl-CoA-dependent enzymes such as GTPSCS and ACSS2 [15,21,22]. In the future, standardized in vitro enzyme activity assays should be established to compare the catalytic efficiency (kcat/Km) of each candidate writer under the same conditions, and these should be combined with metabolic flux analysis, site-resolved stoichiometry, and genetic loss-of-function models to assess the physiological weight of each writer [4,22,74].

Beyond the classic KAT, AARS, and HDAC families, recent studies have identified additional moonlighting enzymes and potential writers across diverse cellular contexts. For instance, NAA10 was identified as the lactyltransferase of NSUN2 at K508 in acidic tumor microenvironments, driving ferroptosis resistance [79]. KAT6B emerged as the most significantly dysregulated lactyltransferase in metabolic liver disease, catalyzing TNFRSF25 K396 lactylation to aggravate ferroptosis [80]. S100A9 and ACAT1 have also been implicated in myocardial ischemia–reperfusion injury and ulcerative colitis as alternative lactylation regulators [81,105]. In prokaryotic systems, the Gcn5-related N-acetyltransferase superfamily member GNAT13 in *Streptococcus mutans* transfers lactyl groups to RpoA (K173), thereby inhibiting biofilm formation [82]. While these candidates are categorized as “Preliminary” or “Candidate” due to limited mechanistic depth compared with p300/CBP, they highlight the expanding enzymatic repertoire of lactylation and warrant further genetic validation.

## 5. Lactylation Erasers, Readers, and Their Crosstalk with Other Post-Translational Modifications (PTMs)

Lactylation is not a static mark unidirectionally determined by writers [106,107]. Its net level is determined by the kinetic balance between writing and erasing [93,94], its functional output is filtered by the recognition efficiency and selectivity of readers, and its informational content is embedded in a PTM network composed of acetylation, methylation, ubiquitination, and RNA modifications [90,108].

### 5.1. Erasers

SIRT family: NAD^+^ dependence directly couples lactylation erasure to energy status [109]. SIRT1, SIRT2, and SIRT3 have all been reported to possess de-lactylase activity [55,110]. From a chemical perspective, SIRT-catalyzed deacylation uses NAD^+^ as a co-substrate and completes amide bond hydrolysis through formation of an ADP-ribosylated acyl intermediate [111]. The difference between lactylated and acetylated substrates lies in the additional hydroxyl group on the α-carbon; how this hydroxyl group affects the catalytic cycle of the SIRT active site still lacks structural biology data [111]. Reported substrates include: SIRT1 delactylates H3K18la [112] and shows eraser activity on the non-histone substrates YAP and PKM2 [110,113]; SIRT3 delactylates PDHA1 in mitochondria [114].

HDAC1-3: Directional competition. Class I HDACs have been reported to possess both de-lactylase and lactylation writing activities [23]. Under basal metabolic conditions, HDAC1-3 may maintain net de-lactylase activity [75,115]; whereas in specific microenvironments with extreme L-lactate accumulation and acetate depletion, the net direction may reverse [65,75]. Validation of this hypothesis requires site-level net flux measurements, and such flux analyses are currently rarely reported in the lactylation field [89,90].

In addition, HDAC8 has been reported to de-lactylate PRMT1, thereby affecting arginine methylation and tumor metastasis [103,116]. The de-lactylase function of prokaryotic CobB has genetic evidence in *E. coli* [24], but the functional correspondence between its mammalian homologs (the SIRT family) and CobB is not direct [117,118,119].

### 5.2. Readers

The Kla readers reported to date include BRG1, DPF2, TRIM33, BRD9, AF9, and ZCWPW2 (Table 2), which recognize lactylated lysine through bromodomains, PHD, YEATS, or PWWP domains, respectively [92,93,94,95,96,97,101,120,121]. AF9 recognizes H3K9la through its YEATS domain, with relatively low affinity, possibly corresponding to a “threshold-sensing mechanism” [65,96]; ZCWPW2 can simultaneously recognize H3K18la and H3K9me3, making it the first lactylation–methylation dual-mark reader [65,97]. In addition, multiple candidates have been reported successively. However, all the newly identified readers require more independent validation, and readers for non-histone Kla remain rarely reported, constraining systematic understanding of lactylation functional output [98,122].

### 5.3. Crosstalk Between Kla and Other PTMs and RNA Modifications

Several mechanistic clues now exist for crosstalk between lactylation and other post-translational modifications, among which the most complete evidence concerns the cascade relationship between lactylation and ubiquitination: after AARS2 catalyzes lactylation of AP-2γ, this modification promotes binding of AP-2γ to TRIM28, thereby enhancing K63-linked ubiquitination and subsequent nuclear translocation [123]. Each step of this ordered lactylation-ubiquitination pathway has clear enzyme–substrate correspondence (AARS2 as the writer, TRIM28 as a component of the ubiquitin E3 ligase complex) [123]. However, whether the lactylation site directly serves as a recognition signal for TRIM28 or merely indirectly exposes the binding region by altering AP-2γ conformation still lacks structural data for discrimination. Notably, the competitive relationship between lactylation and ubiquitination has been independently validated across multiple substrates. In colorectal cancer, lactylation of S100A8 at K23/K36 directly weakens the interaction between S100A8 and the E3 ubiquitin ligase RNF5, thereby inhibiting RNF5-mediated ubiquitination and proteasomal degradation and promoting CRC progression [124]. In cervical cancer, HNRNPU K181 is simultaneously regulated by lactylation and NAA50-mediated acetylation, with the two modifications showing a competitive antagonistic relationship—lactylation reduces HNRNPU ubiquitination and enhances its stability, whereas acetylation produces the opposite effect, thereby constituting a dynamic post-translational modification switch [125]. In colorectal cancer, lactylation of NOL6 at K54 inhibits its ubiquitination and proteasomal degradation, thereby stabilizing the protein. This stabilization subsequently upregulates c-Myc transcription via STAMBP-mediated YY1 deubiquitination. Together, these events establish a positive feedback loop comprising NOL6, STAMBP, YY1, and Myc [126]. In hepatocellular carcinoma, the deubiquitinase JOSD1 and the lactylation writer AARS1 coordinately regulate a ubiquitination–lactylation competitive switch at PGAM1 K251, stabilizing PGAM1 and enhancing glycolytic flux, thereby weakening CD8^+^ T cell infiltration and function [127]. In addition, AlaRS-mediated PARP1 lactylation (K249/K667) overlaps with known ubiquitination sites, suggesting that lactylation may protect PARP1 from degradation by competitively occupying ubiquitination sites, promoting HNSCC progression [128]. The above studies indicate that the competitive relationship between lactylation and ubiquitination has become one of the most common paradigms in this PTM crosstalk field. At the interface between lactylation and methylation, ZCWPW2, as the first reported lactylation–methylation dual-mark reader, can simultaneously recognize H3K18la and H3K9me3 [97], providing a concrete molecular carrier for signal integration between the two modifications. However, whether its recognition occurs as cis-binding on the same histone tail or as trans-binding to different nucleosomes, and whether the dual mark produces synergistic enhancement, remain to be further elucidated. Notably, this study further revealed that the ZCWPW1–ZCWPW2 complex can enhance the function of key lactylation regulators LDHA and EP300, thereby promoting lactylation of recombination-related proteins and stabilizing their abundance; ZCWPW2 deficiency leads to impaired meiotic homologous chromosome synapsis and defective DNA double-strand break repair, suggesting that this dual-mark recognition has an irreplaceable function in physiological processes [97]. More distant crosstalk occurs between lactylation and RNA modifications: histone lactylation (such as H3K18la) can regulate the transcriptional expression of m^6^A-related enzymes (such as METTL3 and YTHDF2) [129,130,131], and METTL3 itself can also be lactylated, affecting its methyltransferase activity [129]. HDAC2-mediated de-lactylation of METTL3 can promote the interaction between METTL3 and Wilms’ tumor-associated protein, increasing m^6^A modification of DNA damage repair-related transcripts and thereby promoting chemoresistance in triple-negative breast cancer [132]. The downstream mechanism by which histone lactylation regulates METTL3 expression has been independently validated in multiple disease models: in sepsis-associated pulmonary fibrosis, LPS-induced histone lactylation upregulates METTL3 expression, and METTL3 recognizes m^6^A-modified PGC-1α mRNA and promotes its degradation, leading to mitochondrial dysfunction and lung fibroblast activation [133]; in ligamentum flavum ossification, histone lactylation regulates METTL3 expression and promotes OLF progression through IGF2BP1-dependent BMP2 m^6^A methylation [134]; in astrocyte activation after intracerebral hemorrhage, H3K18la upregulates METTL3 expression and m^6^A levels, and METTL3 regulates LCN2 m^6^A modification and upregulates its expression, mediating A1-type astrocyte activation [135]. Emerging evidence also points to the regulation of m^6^A readers by lactylation. Specifically, AARS2-mediated lactylation of YTHDF3 enhances its stability, driving cisplatin resistance in bladder cancer via m^6^A-dependent KDM6B degradation and the subsequent relief of CDKN1A inhibition [136]. These findings outline a potential metabolic-epigenetic cascade that links L-lactate and histone lactylation to the transcriptional regulation of m^6^A enzymes and downstream RNA modification. While conceptually compelling, this model currently relies heavily on correlative evidence. A critical unresolved question is whether METTL3 lactylation directly alters its catalytic activity, or whether the observed effects are secondary to changes in its expression levels. Addressing this distinction will require rigorous causal validation in future studies.

### 5.4. Context-Dependent Integrated Model

**Figure 4:**
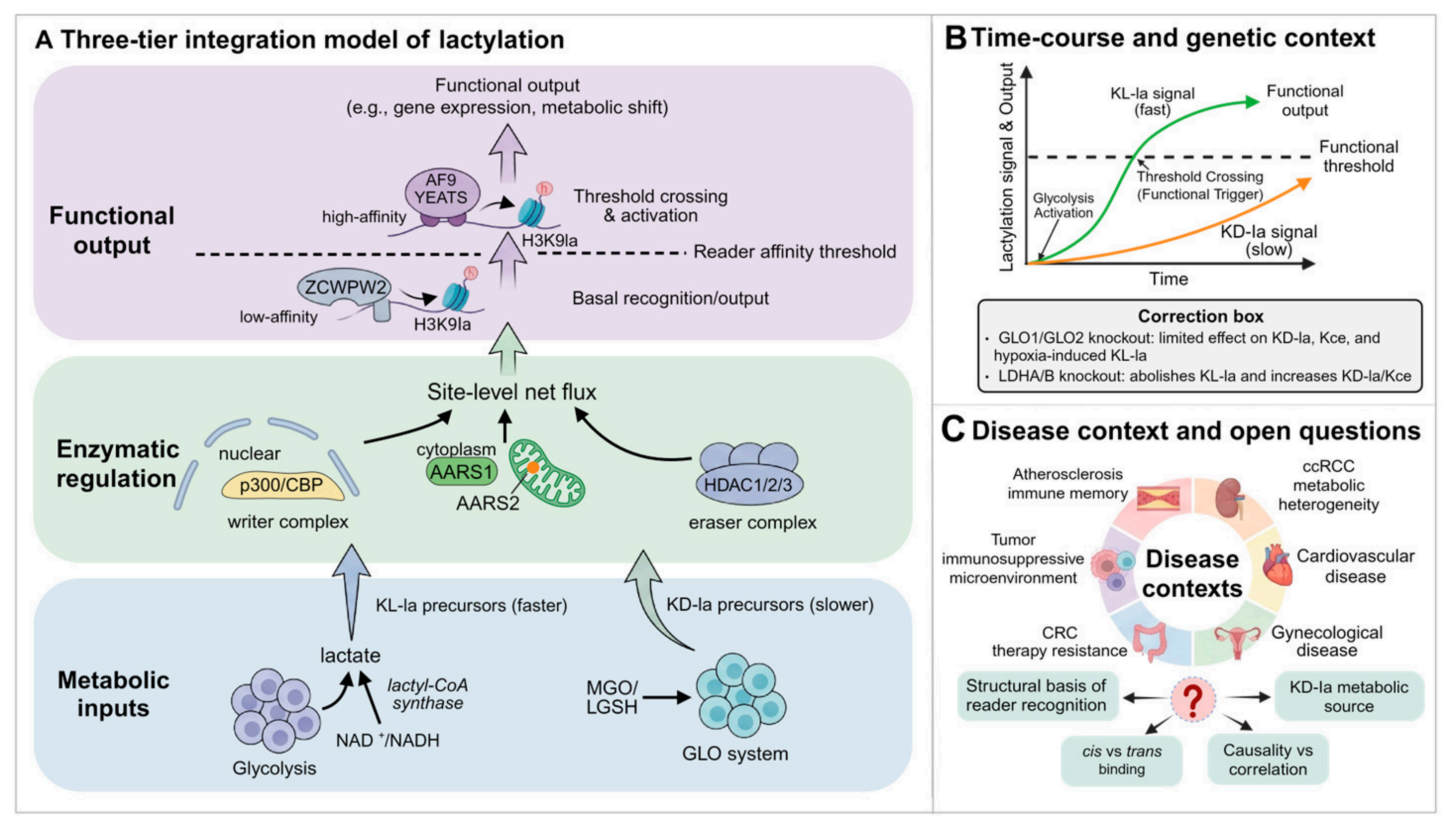
A three-tier integration model of lactylation, time-course dynamics, and disease contexts. (**A**) The three-tier integration model of lactylation, spanning metabolic inputs, enzymatic regulation, and functional outputs. (**B**) Time-course and genetic context of KL-la and KD-la signals, illustrating threshold crossing and functional triggers. (**C**) Disease contexts associated with lactylation and current open questions in the field. The letter "h" within the nucleosome represents a histone molecule carrying the specific modification. Created in BioRender. Zhang, S. (2026) https://BioRender.com/uwcsqhu accessed on 20 October 2026.

Integrating the aforementioned evidence on writers, erasers, readers, and crosstalk, lactylation regulation can be conceptualized as a three-layer architecture (Figure 4) [13,48,70]. The metabolic input layer uses L-lactate concentration, lactyl-CoA synthetase activity, NAD^+^/NADH ratio, and MGO/LGSH levels as core variables; these factors jointly determine the relative weights of the KL-la and KD-la pathways: when glycolysis is active and the glyoxalase system is normal, KL-la predominates, whereas when glyoxalase function is impaired, the contribution of KD-la increases [9,52,137,138]. The core of the enzymatic regulation layer is not the single identity of any writer or eraser, but rather the site-level net flux. Within this layer, the role of p300/CBP may be concentrated in chromatin microenvironments where nuclear lactyl-CoA can be locally enriched; AARS1/2 function on specialized substrates in the cytoplasm and mitochondria; and the directionality of HDACs depends on the local L-lactate/acetate concentration ratio and complex conformational constraints, with the latest evidence indicating that HDAC1/2/3 themselves can act as lactylation writers to catalyze Kla formation [21,23,71]. The functional output layer is determined by the presence or absence of readers and their binding affinity thresholds. Low-affinity readers (such as the YEATS domain of AF9 for H3K9la) may be activated only under high-lactylation states, constituting a “metabolic signal threshold gate,” whereas high-affinity or dual-mark readers (such as ZCWPW2) may function continuously even at basal levels [96,97]. Based on this model, a testable prediction can be proposed: in the early phase of glycolytic activation, the rise in KL-la should precede any detectable functional output, because the binding threshold of readers must be crossed; however, recent studies have raised an important revision regarding the relationship between GLO2 loss and KD-la accumulation—knockout of GLO1 and GLO2 has only limited effects on KD-la, Kce, and hypoxia-induced KL-la, whereas LDHA/B knockout completely abolishes KL-la induction and increases KD-la and Kce levels [52]. This finding suggests that GLO2 loss may not drive KD-la accumulation as significantly as previously expected, and the metabolic source and regulatory mechanism of KD-la still need further clarification [52]. Nevertheless, this temporal differentiation hypothesis of KL-la as a “fast signal” and KD-la as a “slow mark” can still serve as a reference framework for subsequent experimental design to distinguish the functional contributions of the two lactylation pathways [9]. In addition, lactylation does not operate in isolation but exhibits extensive synergistic or competitive crosstalk with acetylation, phosphorylation, ubiquitination, and other post-translational modifications; these interactions further increase the complexity of the integrated model in different cellular contexts [139]. In disease contexts, the lactylation integration model has been preliminarily validated in directions such as atherosclerotic immune memory, metabolic heterogeneity of clear cell renal cell carcinoma, cardiovascular disease, gynecological diseases, colorectal cancer treatment resistance, and the tumor immunosuppressive microenvironment, suggesting that this three-layer architecture has general applicability across tissues and diseases [71,115,137,138,140,141].

## 6. Therapeutic Implications

### 6.1. Upstream Metabolic Intervention

Upstream metabolic intervention indirectly reduces lactylation by lowering L-lactate availability, targeting glucose uptake, glycolysis, L-lactate production, and efflux (Table 3). Among LDH inhibitors, FX11 reduces L-lactate and downregulates histone lactylation in SCLC-A models at 75–105 μM [142]; oxamate lowers H3K18la in glioblastoma, downregulates PD-1/Tim-3 on CAR-T cells, and prevents exhaustion [143]; stiripentol is already used for Dravet syndrome but remains preclinical in oncology [144]. The therapeutic window for LDH inhibition may be narrow [145]. The MCT1 inhibitor AZD3965 in a phase I trial (NCT01791595) confirmed safety in 11 patients with relapsed/refractory DLBCL and Burkitt lymphoma, with 1 complete response (lasting 15 months), 1 stable disease, and 8 non-responders; the low response rate highlights the need for marker-based selection [146]. The MCT4 inhibitor syrosingopine reduces L-lactate efflux in breast cancer, increases M1 macrophages and NK cells, and decreases Tregs [147]. 2-DG is limited by non-selective toxicity [148]; the GLUT1 inhibitor BAY-876 delivered intratumorally delays glioblastoma progression and prolongs survival, but metabolic compensation mechanisms have not been evaluated [149]. Upstream strategies offer the advantage of global L-lactate reduction, but still face challenges including metabolic toxicity, compensation, and a narrow therapeutic window, requiring precise vulnerability identification or combination therapy.

### 6.2. Targeting Writers

Directly targeting writers offers enzyme-specific intervention, but selectivity varies markedly [85]. p300/CBP inhibitors are the most studied: CCS1477 has entered phase I/II (NCT04880062) in combination with anti-PD-1 [156]; A485 inhibits p300 in small cell lung cancer, blocks H4K5la, and restores MHC-I presentation [142]. However, p300/CBP catalyzes both acetylation and lactylation, and compounds such as C646 inhibit both equally, making the shared enzymatic machinery a major source of interference [101]. AARS1/2 inhibitors are more specific (not involved in acetylation): kukoamine A (an AARS2 inhibitor) synergizes with PD-1 blockade in hepatocellular carcinoma [123]; irinotecan inhibits AARS1-mediated BLM K24la and alleviates anthracycline resistance [157]. Recent reviews have further revealed the competitive mechanism by which p300/CBP catalyzes acetylation versus lactylation, systematically evaluated the clinical translation potential of CCS1477 (in phase I/II trials) and A-485, and proposed that PROTAC degraders (such as dCBP-1 and CBPD-409) could degrade p300/CBP protein through the ubiquitin-proteasome pathway, potentially overcoming resistance to traditional catalytic inhibitors [30]. In addition, the novel mechanism of AARS1/2 as acyl-CoA-independent lactylation “writers” has been systematically summarized in independent reviews, covering their functional transition from protein synthesis to lactylation-driven tumorigenesis, as well as multiple strategies for targeting AARS1/2 (including enzyme activity inhibition, alanine antagonism, and blockade of nuclear translocation) [85].

### 6.3. Targeting Readers

Targeting readers blocks lactylation functional output without interfering with metabolism or writer enzymes, offering higher specificity [158]. The mechanism of BRD9 is the most deeply studied: its bromodomain recognizes H3K18la with weak and transient affinity, forming a threshold gate [95]. In liver cancer, H3K18la recruits ncBAF to enhancers/promoters via BRD9, promoting chromatin accessibility and driving oncogenic transcription of SPARC, TMEM64, ANGEL1, SCARB1, and others [159]; H3K18la levels correlate with poor prognosis, and glycolysis inhibition or BRD9 targeting can displace BRD9 and inhibit proliferation [160]. BRD9 is a key mediator between metabolic state and oncogenic transcription, but BRD9 intervention remains preclinical, and selectivity and division of labor among multiple readers still require validation [90,161].

### 6.4. Combination Therapeutic Strategies and Biomarkers

Lactylation-driven immune evasion and drug resistance provide a rationale for combination therapy, with the logic of simultaneously blocking metabolic-epigenetic and immune/targeted pathways to produce synergy [162,163]. In glioblastoma, H3K18la upregulates CD39, CD73, and CCR8, and oxamate combined with CAR-T prevents exhaustion [143,164]; in renal cell carcinoma, zingerone A inhibits LDHA, lowers L-lactate, and disrupts the HIF-1α/VEGF/VEGFR2 axis, enhancing sunitinib efficacy [165]. Regarding biomarkers: a lactylation gene model (NLRP12) shows prognostic potential [166]; H3K18la levels can predict anti-PD-1 efficacy and guide stratification [167,168]. Future studies should test whether integrating lactylation site maps with metabolomics and circulating biomarkers can improve patient stratification [19,169].

## 7. Conclusions and Outlook

Lactylation is not a “metabolic variant” of acetylation. The coexistence of L- and D-chiral forms, the parallel operation of enzymatic and non-enzymatic pathways, and the drastic fluctuation of substrate concentrations with metabolic state mean that it resembles a dynamic chemical picture recording cellular metabolic status rather than a pre-encoded set of signaling rules. Among these, L-lactylation is driven by glycolysis and can be dynamically regulated by specific erasers and readers; D-lactylation is mainly produced non-enzymatically via the methylglyoxal pathway, has extremely low erasure efficiency, and its reading mechanisms are almost unknown. If the two are indiscriminately lumped together as “lactylation,” this may cause persistent misinterpretation in functional studies. Likewise, the lactylation field has not yet discovered a truly dedicated writer enzyme; all candidates are moonlighting participants in other metabolic or acylation pathways. Therefore, the more meaningful question is no longer “whether a given enzyme is a writer,” but “under what conditions, at which sites, and with what flux does the enzyme make a detectable contribution to site-specific lactylation.” This shift moves the research focus from identity assignment to quantitative attribution.

The establishment of enantiomer-resolved technology in 2024 was an important watershed: it revealed that much of the data obtained over the previous five years using antibodies marketed as pan-Kla (in practice, anti-L-lactylation antibodies) actually represented mixed signals of L-lactylation, D-lactylation, and carboxyethylation. Most commercial anti-lactylation antibodies available before 2024 were raised against L-lactyl peptides and are therefore best described as anti-L-lactylation antibodies; however, their enantiomer specificity had not been rigorously validated by chiral resolution. They should not be regarded as validated pan-Kla reagents. Conclusions based on those antibodies should therefore be re-evaluated with isomer-resolved methods. Yet antibodies marketed as pan-Kla (in practice, anti-L-lactylation antibodies) remain the default tool in many laboratories, and isomer resolution has not yet become a routine analytical mode. What the field truly lacks is not candidate enzymes, but an integrated framework—moving from “who can catalyze” to “when do they contribute,” and from in vitro enzyme activity to intracellular flux attribution. Achieving this transition will require coordinated breakthroughs in flux measurement tools, identification of non-histone readers, and site-specific intervention approaches.

## Figures and Tables

**Table 2:** Confirmed and candidate Kla readers.

Reader	Modification(s) and Recognition Domain	Key Biological Functions	Key Evidence/Methodology	Evidence Level	References
BRG1	H3K18la; bromodomain	Promotes MET during iPSC reprogramming and activates core pluripotency genes (Oct4, Sall4, Cdh1, Cldn3/4, Epcam)	AlphaFold modeling; ChIP-qPCR; Brg1 overexpression and knockout in early reprogramming; Dux overexpression-induced Brg1 accumulation	Confirmed	[92]
DPF2	H3K14la; dual PHD zinc-finger domains (PHD1–PHD2)	Drives cervical cancer tumorigenesis through oncogene activation (SEMA5A, ROCK1) and supports cell cycle progression and anti-apoptotic signaling	Multivalent photoaffinity probe with quantitative proteomics (PXD051995); ITC; CUT&Tag (GSE267107, GSE267109); structure-guided mutagenesis; RNA-seq	Confirmed	[93]
TRIM33	H3K18la, H4K5la, H4K8la, H4K12la; PHD-bromodomain cassette	Promotes macrophage M1→M2 polarization; attenuates inflammatory gene transcription; acts as dominant reader in the hypoxic tumor core	AlphaScreen; ITC; protein-detected NMR (^1^H/^15^N, ^13^C/^15^N); HA-TRIM33β immunoprecipitation under L-lactate induction; molecular modeling; E981A mutagenesis	Confirmed	[94]
BRD9	H3K18la; bromodomain	Recruits ncBAF to active enhancers and promoters in HCC; drives oncogenic transcription (SPARC, TMEM64, ANGEL1, SCARB1); H3K18la levels correlate with poor prognosis	NMR solution structures; multi-omics (RNA-seq GSE314155, ChIP-seq, ATAC-seq); glycolytic inhibition with 2-DG; BRD9 targeting; p300 and HDAC inhibition	Confirmed	[95]
AF9	H3K9la; YEATS domain	Forms an AF9–KLF2 positive feedback loop driving metabolic reprogramming, TGF-β signaling, and immune evasion in luminal breast cancer	Screening of 12 reader proteins; biochemical binding assays; crystal structures (PDB: 8Z73, 9IM4); spatial transcriptomics; scRNA-seq	Confirmed	[96]
ZCWPW2	H3K18la and H3K9me3 (dual histone mark reader); PWWP domain plus CW domain	Regulates meiotic recombination; deficiency causes homologous chromosome synapsis defects and DSB repair defects in humans and mice; stabilizes recombination-associated proteins through lactylation	Lactylomic proteomics (PXD068838); meiotic recombination assays; mouse and human genetic models; LC–MS/MS of ZCWPW2-associated proteins; crystal structure (PDB: 4Z0R)	Confirmed	[97]
BRD4	H4K8la (putative); BRD4 K317la (self-modification); bromodomain	Required for L-lactate-induced CSC dedifferentiation; promotes BET inhibitor resistance; BRD4 inhibition reduces CRC tumor growth and liver metastasis	Functional studies with BRD4 inhibition; bioinformatics analysis; self-lactylation site mapping	Candidate	[99]
RRP8	Candidate Kla reader; methyltransferase domain	eNoSC complex component and known H3K9me2/3 reader; candidate chromatin reader for Kla	Semisynthetic nucleosome probes combined with Co-IP and LC–MS/MS	Candidate	[100]
DNMT3A	H3K18la, H3K27la (speculative); DNMT domain	Silences tumor suppressor genes and contributes to drug resistance; acts as a L-lactate transporter promoting HIF-1α lactylation	Review-level inference; PKM2–DNMT3A–SMAD2 axis study	Candidate (speculative)	[101,102]
PRMT1	K134/K145la (self-modification); PRMT domain	Promotes vimentin asymmetric dimethylation and tumor metastasis; participates in a PRMT1–LDHA–STAT3 feedback axis	shRNA screening of KAT7 and HDAC8; functional invasion assays	Candidate	[103]
BRD7	Candidate Kla reader; bromodomain	Known H3K14ac reader with a potential role in chromatin remodeling	Semisynthetic nucleosome probes combined with Co-IP and LC–MS/MS	Candidate	[104]

Note: Confirmed: Direct experimental evidence from original research, including structural, biophysical, or quantitative binding data, supporting the reader’s specific recognition of Kla marks; Candidate: Supported by screening, functional, or review-level evidence, but lacking direct structural or quantitative binding validation for Kla recognition; Candidate (speculative): Proposed based on review-level inference or indirect evidence only; direct experimental validation is required.

**Table 3:** Therapeutic strategies targeting lactylation pathways.

Layer	Strategy	Representative Drug/Molecule	Target	Lactylation Specificity	Evidence Level	Clinical Stage	Key References
Upstream lactate metabolic regulation	LDH inhibition	Oxamate, stiripentol, FX11	LDHA	Low (global L-lactate reduction)	Medium	Preclinical; stiripentol already clinical for epilepsy	[13]
	MCT1 inhibition	AZD3965	MCT1	Low (intracellular L-lactate may increase)	Medium	Phase I completed; phase II ongoing	[150]
Writer inhibition	p300/CBP inhibition	CCS1477 (Inobrodib), A-485, C646	p300/CBP	Low (also inhibits acetylation)	High	CCS1477 in phase I/II (NCT03568656, NCT04068597)	[70,101]
	AARS1/2 inhibition	Kukoamine A delivered by nanocarrier	AARS2	Medium (not involved in acetylation)	Low to medium	Preclinical	[123]
	KAT2A inhibition	MB-3	KAT2A	Medium (KAT2A can catalyze lactylation)	Low	Preclinical	[151]
Reader targeting	Small-molecule reader inhibition	BI-9564	BRD9	Undefined (Kla/Kac selectivity not quantified)	Low	Preclinical	[95]
	PROTAC-mediated reader degradation	CFT8634	BRD9	Undefined	Low	Phase I (SMARCB1-perturbed or SMARCB1-null tumors)	[152,153]
Eraser regulation	Delactylase activation (SIRT)	SRT2104 (SIRT1 activator); honokiol (SIRT3 activator)	SIRT1, SIRT3	Medium (SIRT also regulates acetylation and succinylation)	Low to medium	Preclinical; SRT2104 has early clinical trials in metabolic disease	[154]
	Delactylase inhibition (HDAC)	TSA, MS-275, vorinostat	HDAC1–3	Low (also affects acetylation; directionality paradox: HDAC inhibition leads to Kla accumulation)	Medium	Preclinical; vorinostat approved for other indications	[154]
	D-lactylation pathway modulation	GLO2 inhibitor DiFMOC-G	GLO2	High (affects only the D-lactylation pathway)	Low	Preclinical	[44]
Combination therapy	Immunotherapy combination with PD-1 blockade	Oxamate or nano-Kukoamine A, each combined with anti-PD-1; AZD3965 combined with anti-PD-1	LDHA, AARS2, MCT1	Low to medium	Medium	Preclinical	[123,155]
	Immunotherapy combination with CAR-T	Oxamate combined with CAR-T	LDHA	Low to medium	Low to medium	Preclinical	[143]
	Vaccine combination	L-lactate or lactylation modulation combined with cancer vaccine	Upstream L-lactate metabolism	Low	Very low	Preclinical	[155]

Note: Lactylation specificity is defined as follows: High, selectively targets the lactylation pathway without affecting other acylation modifications (e.g., acetylation); Medium, predominantly affects lactylation with limited cross-reactivity with other modifications, or directly targets a specific lactylation writer/eraser; Low, indirect modulation (e.g., global L-lactate reduction) or significant cross-inhibition of other modifications (e.g., acetylation); Undefined, selectivity for lactylation over other modifications has not been quantified. Evidence levels are defined as follows: High, multiple independent studies plus clinical data; Medium, multiple preclinical studies plus partial clinical validation; Low to medium, limited preclinical studies plus preliminary mechanistic validation; Low, a single or few preclinical studies; Very low, conceptual speculation.

## Data Availability

No new data were created or analyzed in this study. Data sharing is not applicable to this article.

## Supplementary Materials

The following supporting information can be downloaded at: https://www.mdpi.com/article/10.3390/molecules31213902/s1, Table S1. Abbreviations.
