# Supplementary material for: Lysine Lactylation Writers: Enzymatic Versus Non-Enzymatic Mechanisms, Isomer Specificity, and Therapeutic Implications
Source: Molecules. 2026 Oct 29;31(21):3902. doi: 10.3390/molecules31213902 (PMC13765021; doi:10.3390/molecules31213902)
Supplement: Supplementary file 1 [file molecules-31-03902-s001.zip › molecules-4609749-supplementary.pdf]

## Table S1. Abbreviations

| Abbreviation | Full term | Abbreviation | Full term |
|---|---|---|---|
| 2-DG | 2-deoxy-D-glucose | AARS1 | aminoacyl-tRNA synthetase 1 |
| AARS2 | aminoacyl-tRNA synthetase 2 | ACSS2 | acetyl-CoA synthetase 2 |
| AF9 | ALL1-fused gene from chromosome 9 protein | AMP | adenosine monophosphate |
| AMPK | AMP-activated protein kinase | AP-2γ | activator protein 2 gamma |
| ATP | adenosine triphosphate | BET | bromodomain and extra-terminal |
| BMP2 | bone morphogenetic protein 2 | BRD4 | bromodomain-containing protein 4 |
| BRD7 | bromodomain-containing protein 7 | BRD9 | bromodomain-containing protein 9 |
| BRG1 | Brahma-related gene 1 | CAR-T | chimeric antigen receptor T cell |
| CBP | CREB-binding protein | CCR8 | C-C chemokine receptor type 8 |
| CD39 | ectonucleoside triphosphate diphosphohydrolase 1 | CD73 | ecto-5′-nucleotidase |
| CD8+ | CD8-positive | CDKN1A | cyclin-dependent kinase inhibitor 1A |
| cGAS | cyclic GMP-AMP synthase | ChIP | chromatin immunoprecipitation |
| CoA | coenzyme A | Co-IP | co-immunoprecipitation |
| CPT2 | carnitine palmitoyltransferase 2 | CRC | colorectal cancer |
| CSC | cancer stem cell | CUT&Tag | cleavage under targets and tagmentation |
| DLBCL | diffuse large B-cell lymphoma | DNMT3A | DNA methyltransferase 3 alpha |
| DPF2 | double PHD fingers 2 | DSB | double-strand break |
| eEF1A2 | eukaryotic elongation factor 1 alpha 2 | EP300 | E1A-binding protein p300 |
| GCN5 | general control nonderepressible 5 | GLO1 | glyoxalase 1 |
| GLO2 | glyoxalase 2 | GLUT1 | glucose transporter 1 |

| | | | |
|---|---|---|---|
| GTPSCS | GTP-specific succinyl-CoA synthetase | H3K14ac | histone H3 lysine 14 acetylation |
| H3K14la | histone H3 lysine 14 lactylation | H3K18la | histone H3 lysine 18 lactylation |
| H3K27la | histone H3 lysine 27 lactylation | H3K9ac | histone H3 lysine 9 acetylation |
| H3K9la | histone H3 lysine 9 lactylation | H3K9me3 | histone H3 lysine 9 trimethylation |
| H4K12la | histone H4 lysine 12 lactylation | H4K5la | histone H4 lysine 5 lactylation |
| H4K8la | histone H4 lysine 8 lactylation | HAT1 | histone acetyltransferase 1 |
| HBO1 | HBO1 acetyltransferase | HCC | hepatocellular carcinoma |
| HDAC | histone deacetylase | HIF-1α | hypoxia-inducible factor 1 alpha |
| HNRNPU | heterogeneous nuclear ribonucleoprotein U | HNSCC | head and neck squamous cell carcinoma |
| IBD | inflammatory bowel disease | IGF2BP1 | insulin-like growth factor 2 mRNA-binding protein 1 |
| iPSC | induced pluripotent stem cell | ITC | isothermal titration calorimetry |
| JOSD1 | Josephin domain-containing protein 1 | KAT | lysine acetyltransferase |
| Kce | N-ε-carboxyethyllysine | KDM6B | lysine demethylase 6B |
| KD-la | D-lactylation | KL-la | L-lactylation |
| Kla | lysine lactylation | KO | knockout |
| LC–MS/MS | liquid chromatography–tandem mass spectrometry | LCN2 | lipocalin 2 |
| LDHA | lactate dehydrogenase A | LDHB | lactate dehydrogenase B |
| LGSH | S-D-lactoylglutathione | MCT1 | monocarboxylate transporter 1 |
| MCT4 | monocarboxylate transporter 4 | MET | mesenchymal-to-epithelial transition |
| METTL3 | methyltransferase-like 3 | MGO | methylglyoxal |
| MHC-I | major histocompatibility complex class I | MRE11 | meiotic recombination 11 |
| $m^6A$ | N6-methyladenosine | NAA50 | N-alpha-acetyltransferase 50 |

| | | | |
|---|---|---|---|
| $NAD^+$ | oxidized nicotinamide adenine dinucleotide | NADH | reduced nicotinamide adenine dinucleotide |
| ncBAF | non-canonical BRG1/BRM-associated factor | NBS1 | Nijmegen breakage syndrome 1 |
| NF-κB | nuclear factor kappa B | NK | natural killer |
| NLRP12 | NLR family pyrin domain containing 12 | NMR | nuclear magnetic resonance |
| NOL6 | nucleolar protein 6 | OE | overexpression |
| OLF | ossification of the ligamentum flavum | p53 | tumor protein p53 |
| PARP1 | poly(ADP-ribose) polymerase 1 | PDB | Protein Data Bank |
| PD-1 | programmed cell death protein 1 | PDHA1 | pyruvate dehydrogenase E1 component subunit alpha |
| PD-L1 | programmed death-ligand 1 | PGAM1 | phosphoglycerate mutase 1 |
| PGC-1α | peroxisome proliferator-activated receptor gamma coactivator 1 alpha | PHD | plant homeodomain |
| PKM2 | pyruvate kinase M2 | PRMT1 | protein arginine methyltransferase 1 |
| PROTAC | proteolysis-targeting chimera | PTM | post-translational modification |
| PWWP | Pro-Trp-Trp-Pro domain | RelA | REL-associated protein, NF-κB p65 subunit |
| RNF5 | ring finger protein 5 | RPA1 | replication protein A1 |
| RRP8 | ribosomal RNA processing 8 | S100A8 | S100 calcium-binding protein A8 |
| SCLC-A | small cell lung cancer subtype A | SIRT | sirtuin |
| SLG | S-D-lactoylglutathione | SMARCB1 | SWI/SNF-related matrix-associated actin-dependent regulator of chromatin subfamily B member 1 |
| STAMBP | STAM-binding protein | SUCLG2 | succinyl-CoA synthetase subunit |
| TBX5 | T-box transcription factor 5 | TCA | tricarboxylic acid |

| | | | |
|---|---|---|---|
| TEAD1 | TEA domain transcription factor 1 | TGF-β | transforming growth factor beta |
| Tim-3 | T-cell immunoglobulin and mucin-domain containing-3 | TIP60 | Tat-interactive protein 60 |
| Treg | regulatory T cell | TRIM28 | tripartite motif-containing 28 |
| TRIM33 | tripartite motif-containing 33 | TSA | trichostatin A |
| TTP | tristetraprolin | VEGF | vascular endothelial growth factor |
| VEGFR2 | vascular endothelial growth factor receptor 2 | YAP | Yes-associated protein |
| YEATS | Yaf9, ENL, AF9, Taf14, Sas5 domain | YTHDF2 | YTH N6-methyladenosine RNA binding protein 2 |
| YTHDF3 | YTH N6-methyladenosine RNA binding protein 3 | ZCWPW1 | zinc finger CW-type and PWWP domain containing 1 |
| ZCWPW2 | zinc finger CW-type and PWWP domain containing 2 | α-tubulin | alpha-tubulin |
